# Supplementary material for: Living-Cell Diffracted X-ray Tracking Analysis Confirmed Internal Salt Bridge Is Critical for Ligand-Induced Twisting Motion of Serotonin Receptors
Source: Int J Mol Sci. 2021 May 17;22(10):5285. doi: 10.3390/ijms22105285 (PMC8157010; doi:10.3390/ijms22105285)
Supplement: Supplementary file 1 [file ijms-22-05285-s001.zip › ed_ijms-1198912-Modified-Supplementary-Material-Final.pdf]

Supplementary Material

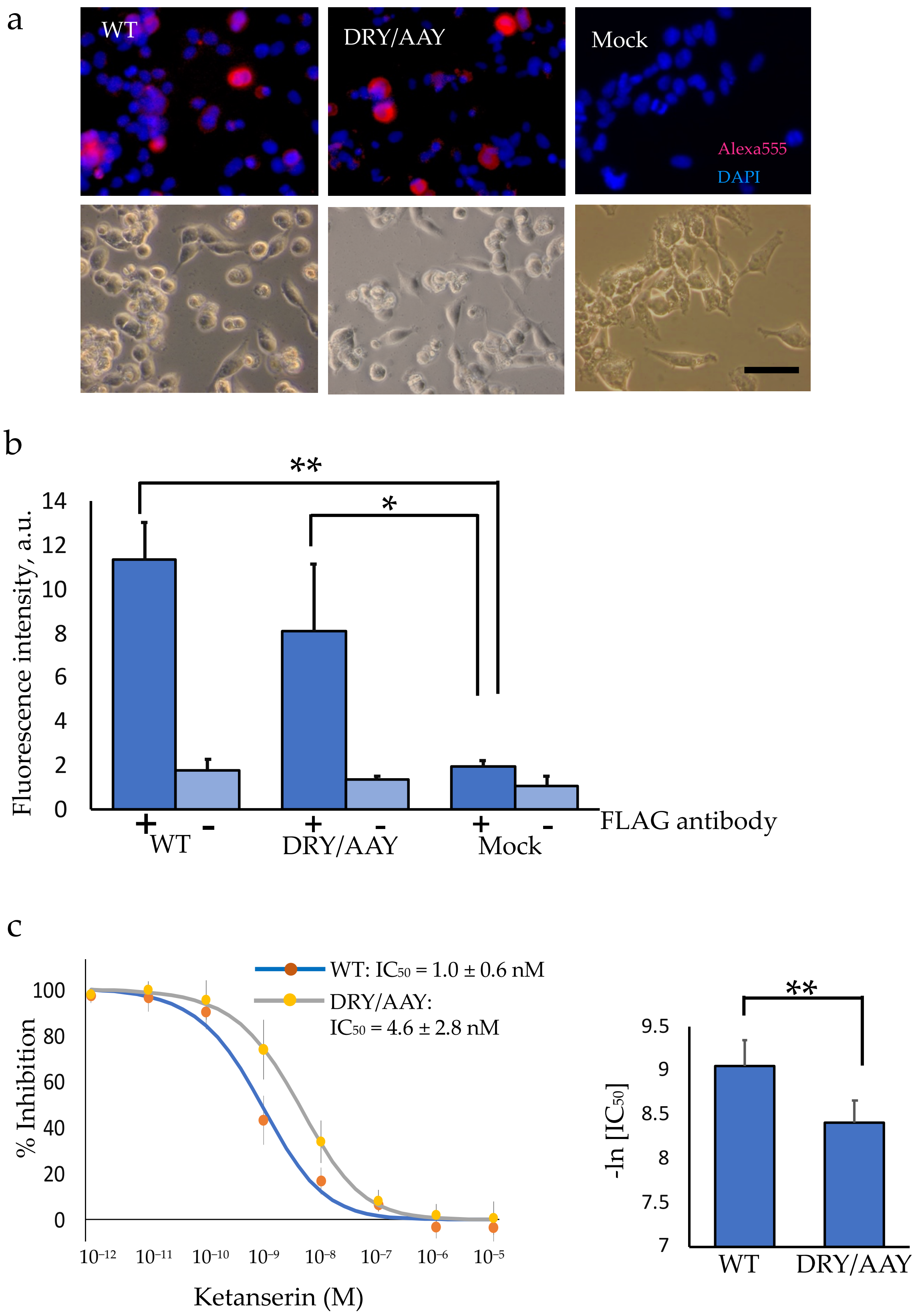

Figure S1. Expression and function of 5-$HT_{2A}R$ used in this experiment. (**a**) Expression of N-FLAG 5-HT_{2A} receptors (WT) and DRY/AAY in HEK293 cells was confirmed by immunocytochemical studies using anti-FLAG antibodies. The cells were stained with monoclonal anti-FLAG M2 antibody (Sigma-Aldrich: F1804) followed by Alexa Fluor 555 anti-mouse IgG (Thermofisher: A31622). Both WT and DRY/AAY expressing cells were positive for FLAG antibodies. About 40% of the cells were positive for Alexa555 in this transient expression, while no positive cells were observed in the mock transfection. Lower panels: corresponding bright-field images. Scale bar, 50μm. (**b**) Live-cell based immunofluorescence assay. Living cells in suspension were labelled with anti-FLAG M2 antibody, followed by Alexa Fluor 488-conjugated secondary antibody (Thermofisher: A11001, Ex/Em = 493/519 nm). The fluorescence intensities were measured with a 96 well plate using the FlexStation 3 system. Both the intensities of WT and DRY/AAY were significantly higher than those of mock transfected cells, suggesting the expression of 5-$HT_{2A}R$ on the cell surface. Experiments without the primary FLAG antibody were presented as a negative control. Data are presented as mean values ± SD (standard deviation) from four different experiments. Two-tailed paired Student *t*-test *p*-values indicate statistical significance ($*p < 0.05$ and $**p < 0.01$). (**c**) Inhibition of α-MS induced calcium influx by ketanserin. HEK 293 cells transfected with WT or DRY/AAY were pretreated with ketanserin at 10^{-5} – 10^{-12} M, then calcium influx by 10μM α-MS was measured using the FlexStation 3 system with the FLIPR Calcium 6 reagent (Molecular Devices Inc.). The IC_{50} were calculated to be 1.0 ± 0.6 nM for WT, and 4.6 ± 2.8 nM for DRY/AAY, respectively. Data are presented as mean ± SD from five different experiments. Right panel; concentration of ketanserin at IC_{50} was analyzed by the Student *t*-test ($**p < 0.01$).

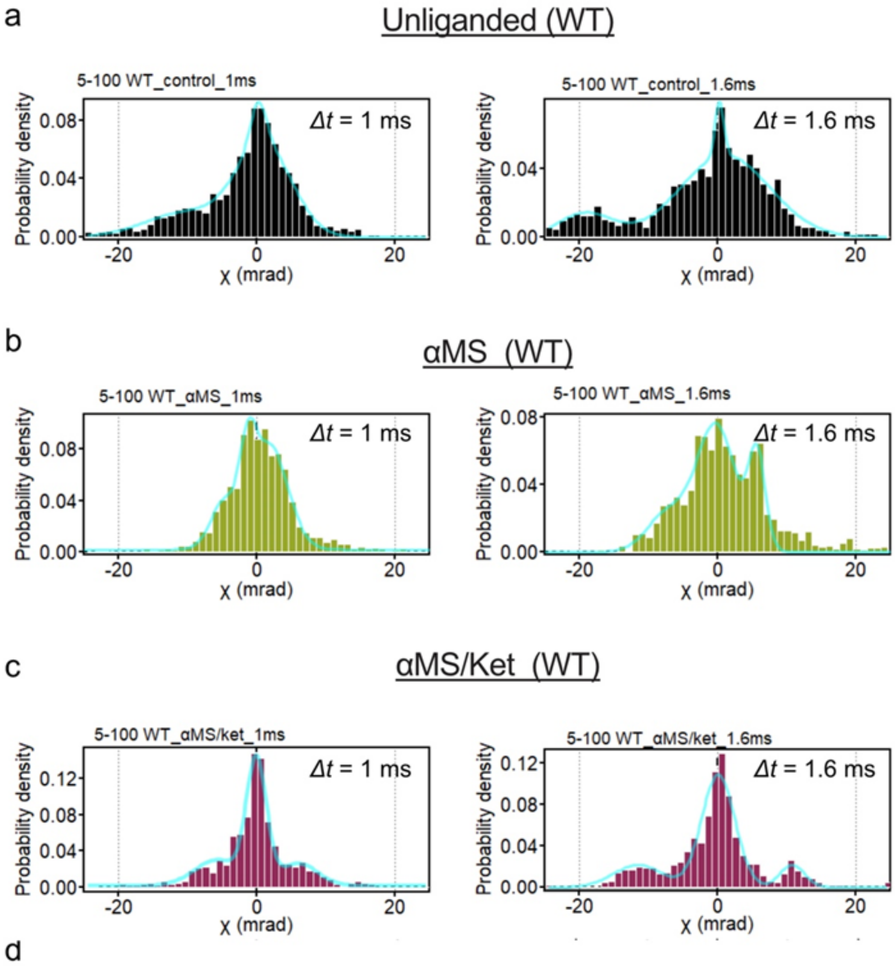

d

		peak 1			peak 2			peak 3		
	time lag Δt	A_0	χ_0	σ_0	A_1	χ_1	σ_1	A_2	χ_2	σ_2
WT	1	0.02	-10.1	6.0	0.03	0.2	1.1	0.06	1.1	4.0
	1.6	0.01	-19.1	3.9	0.03	0.2	0.6	0.05	1.1	6.5
α MS	1	0.04	-4.7	2.0	0.05	-1.3	1.1	0.08	1.8	2.7
	1.6	0.02	-7.2	3.0	0.08	-0.2	2.8	0.06	5.7	1.2
α MS/Ket	1	0.03	-5.7	3.3	0.14	-0.1	1.4	0.02	6..0	2.8
	1.6	0.02	-11.5	3.5	0.11	0.1	2.4	0.02	10.8	1.8

Figure S2. Three Gaussian curve fitting of distribution of angular displacement for wild type 5-HT2AR. (**a–c**) Distribution of the absolute angular displacement of the wild-type 5-HT2AR over an interval time t (left panels, Δt = 1 ms, and right panels, Δt = 1.6 ms) fitted by three Gaussian curves. (**d**) Statistical parameters for fitted curves. Curves were fitted with the following equation, where A represents amplitude, χ represents angular displacement, and σ represents standard deviation.

$$f(\chi) = A_0 \exp(-\frac{(\chi - \chi_0)^2}{2\sigma_0^2}) + A_1 \exp(-\frac{(\chi - \chi_1)^2}{2\sigma_1^2}) + A_2 \exp(-\frac{(\chi - \chi_2)^2}{2\sigma_2^2})$$

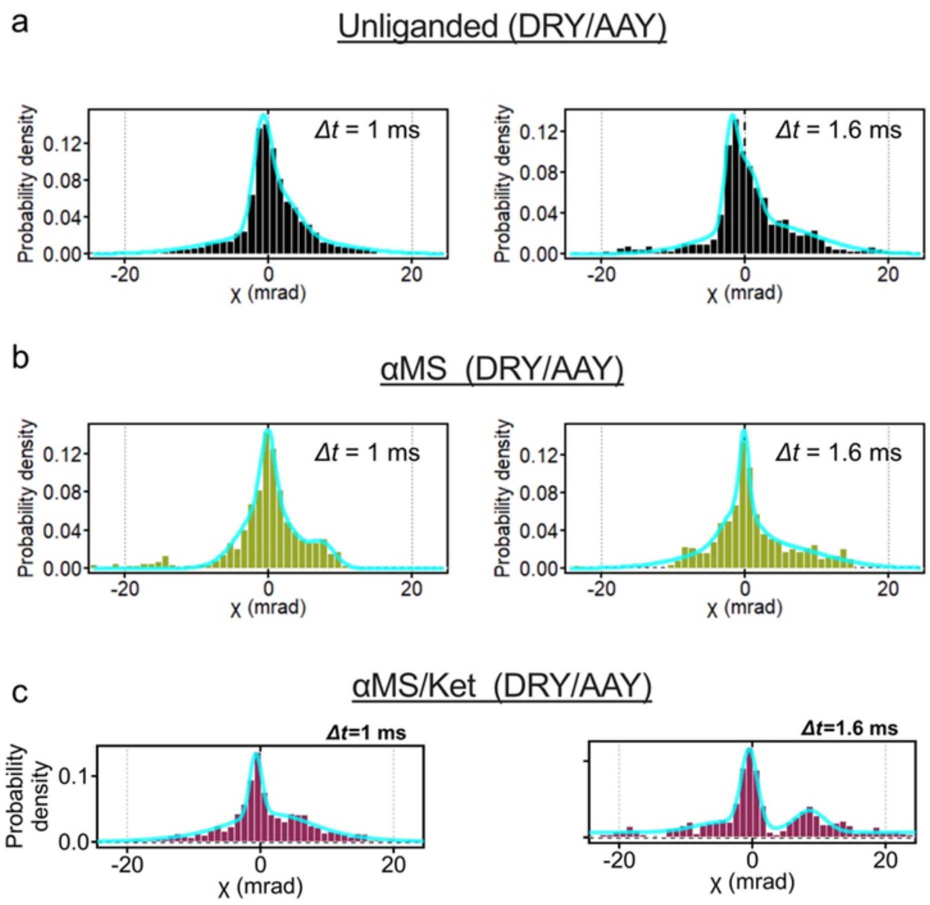

d

	time lag Δt	peak 1 A_0	peak 1 χ_0	peak 1 σ_0	peak 2 A_1	peak 2 χ_1	peak 2 σ_1	peak 3 A_2	peak 3 χ_2	peak 3 σ_2
DRY/AAY	1	0.10	-0.9	1.2	0.02	0.4	8.1	0.04	1.7	2.4
	1.6	0.09	-1.9	0.9	0.06	0.2	1.6	0.03	2.6	7.1
α MS	1	0.07	-0.3	3.6	0.08	-0.1	1.0	0.02	7.6	1.7
	1.6	0.04	-0.8	2.3	0.08	-0.1	0.6	0.03	1.8	7.9
α MS/Ket	1	0.10	-0.7	0.6	0.02	0.7	1.1	0.03	1.6	2.0
	1.6	0.10	-0.5	0.7	0.01	-4.1	0.5	0.03	1.7	1.0

Figure S3. Three Gaussian curve fitting of distribution of angular displacement for DRY/AAY mutant 5-HT_{2A}R. (**a–c**) Distribution of the absolute angular displacement of the DRY/AAY mutant 5-HT_{2A}R over an interval time t (left panels, Δt = 1 ms, and right panels, Δt = 1.6 ms) fitted by three Gaussian curves. (**d**) Statistical parameters for fitted curves.

Table S1. Parameters of MSD curves for wild type 5-$HT_{2A}R$. The MSD curves were fitted by the following function: $\delta^2(t) = D_\alpha t^\alpha + 2\beta^2$ on the range of 0–4 ms. D_α is the anomalous diffusion constant, a non-linear relationship to time, α called subdiffusion ($1 > \alpha > 0$) or superdiffusion ($\alpha > 1$) and β called measurement error.

WT theta

	Ligand-free	αMS	αMS/ket
D_α ($mrad^2/ms$)	5.57	3.31	2.64
α	0.99	0.84	0.46
β^2 ($mrad^2$)	0.003	−0.001	0.003

WT kai

	Ligand-free	αMS	αMS/ket
D_α ($mrad^2/ms$)	66.6	32.2	37.8
α	1.67	1.47	0.97
β^2 ($mrad^2$)	0.889	1.016	0.002

Table S2. Parameters for single Gaussian fitting. The probability density was fitted by the Gaussian function: $f(x) = a \exp(-(x-\mu)^2/2\sigma^2)$.

WT

	Time interval (ms)	a	μ	σ^2
Ligand free	1	0.08	0.54	2.6
	1.6	0.05	0.98	4.2
α-MS	1	0.10	0.08	2.7
	1.6	0.07	0.36	4.0
α-MS/ket	1	0.14	−0.18	1.3
	1.6	0.11	0.05	1.7

DRY/AAY

	Time interval (ms)	a	μ	σ^2
Ligand free	1	0.13	−0.14	1.8
	1.6	0.11	−0.64	1.8
α-MS	1	0.12	−0.20	1.6
	1.6	0.10	−0.30	1.9
